# CoQ_10_ oxidoreductases in ferroptosis and cancer: redox regulation and therapeutic opportunities

**DOI:** 10.1038/s12276-026-01736-w

**Published:** 2026-06-03

**Authors:** Jumi Lee, Inhwan Yoo, Minseok Kim, Hyeonseok Kim, Namgyu Lee

**Affiliations:** 1 Department of Biomedical Science & Systems Biology, Dankook University, Cheonan, Republic of Korea; 2 Department of Microbiology and Biotechnology, Dankook University, Cheonan, Republic of Korea

**Keywords:** Biochemistry, Cancer, Cell biology

## Abstract

Coenzyme Q_10_ (CoQ_10_) is a highly conserved lipophilic redox cofactor that mediates electron transport in mitochondria and protects cells against oxidative stress. Increasing evidence has revealed that CoQ_10_ and its associated oxidoreductases serve as critical regulators of ferroptosis—an iron-dependent, lipid peroxidation-driven form of cell death. Here we provide an updated overview of the canonical CoQ_10_ biosynthetic pathway and the established functions of key CoQ_10_ oxidoreductases. We highlight emerging insights into ferroptosis-suppressive roles of enzymes such as FSP1, DHODH and SQOR, which act by regenerating ubiquinol (CoQ_10_H_2_, reduced). In addition, we discuss newly identified CoQ_10_-related enzymes and their mechanistic contributions to ferroptosis regulation. By integrating perspectives from biochemistry, metabolism and cancer biology, this review positions CoQ_10_ oxidoreductases as promising targets for ferroptosis-based cancer therapies.

## CoQ10 enzymes regulate ferroptosis in cancer cells

Ferroptosis, an iron-dependent form of cell death, emerges as a promising target for cancer therapy, particularly in aggressive tumors such as triple-negative breast cancer. This review explores the role of coenzyme Q10 (CoQ_10_) and its oxidoreductases in ferroptosis regulation, highlighting a therapeutic opportunity. The authors detail the CoQ_10_ biosynthesis pathway and its role in maintaining cellular redox balance, emphasizing enzymes such as FSP1, DHODH and SQOR that reduce CoQ_10_ to its antioxidant form, ubiquinol. These enzymes help to protect cells from lipid peroxidation, a key step in ferroptosis. Notably, targeting the CoQ_10_ redox cycle offers a potential strategy for cancer therapy, as it may lower the threshold for ferroptotic cell death in tumors. Future research should focus on understanding CoQ_10_ distribution across cellular compartments and developing biomarkers to identify tumors dependent on CoQ_10_ oxidoreductases, paving the way for targeted cancer treatments.

This summary was initially drafted using artificial intelligence, then revised and fact-checked by the author.

## Introduction

Ferroptosis is an iron-dependent form of regulated cell death driven by the uncontrolled accumulation of lipid peroxides^1^. As advanced tumor types such as triple-negative breast cancer (TNBC), drug-tolerant persister cells and metastatic cancer cells are particularly vulnerable to ferroptosis, induction of ferroptosis has emerged as a promising therapeutic strategy^2–5^. In addition, conventional treatments such as radiation therapy and chemotherapeutic agents can promote ferroptosis, thereby increasing ferroptosis sensitivity and ultimately enhancing the efficacy of existing anticancer therapies^6,7^.

**Fig. 1:**
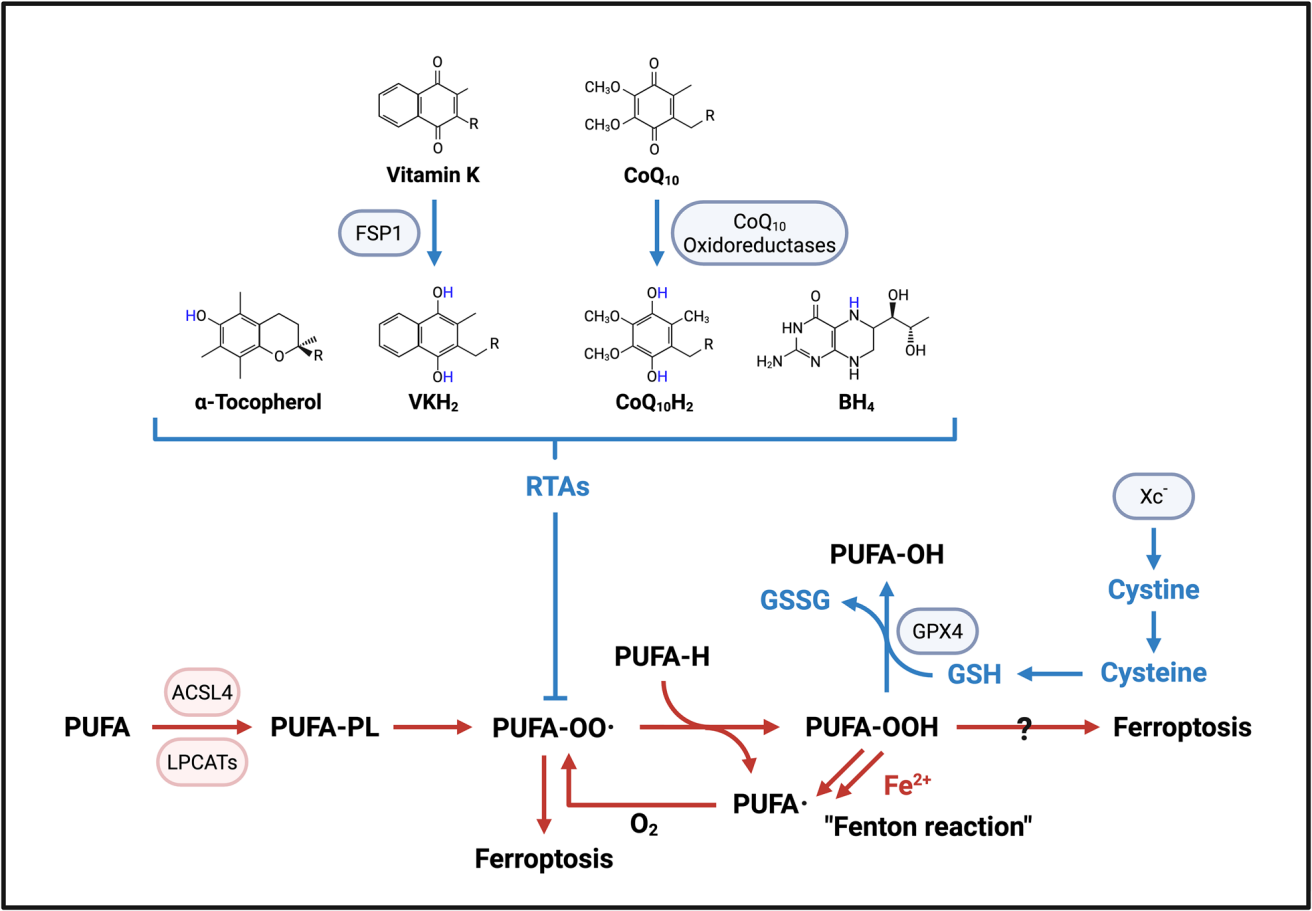
RTA systems that block PUFA lipid peroxidation and prevent ferroptosis. Processes driving ferroptosis are shown in red, whereas protective mechanisms are shown in blue. PUFA-containing phospholipids (PUFA-PL) undergo peroxidation to form phospholipid peroxyl radicals (PUFA-OO·), which propagate lipid damage and drive ferroptosis. RTAs such as α‑tocopherol, ubiquinol (CoQ_10_H_2_), dihydro‑vitamin K (VHK_2_) and BH_4_ neutralize PUFA‑OO·, terminating the chain reaction. GPX4 reduces phospholipid hydroperoxides to nontoxic forms using GSH. CoQ_10_ oxidoreductases regenerate reduced RTAs (for example, CoQ_10_H_2_ and VHK_2_), sustaining antioxidant defense and inhibiting ferroptosis.

Since its initial characterization, a central theme that has emerged is that ferroptosis represents a fundamental metabolic vulnerability—one that arises when antioxidant systems fail to detoxify lipid hydroperoxides (LOOH)^1,8,9^. Among these systems, glutathione (GSH) peroxidase 4 (GPX4) plays a dominant role by converting LOOH into nontoxic lipid alcohols (LOH) using GSH as an electron donor^10^. However, unbiased genetic screens and biochemical studies have demonstrated that GPX4 is not the sole defense against ferroptosis. Cells also harbor multiple parallel, GPX4-independent antiferroptotic pathways, many of which rely on endogenous radical-trapping antioxidants (RTAs) that directly quench lipid radicals (Fig. 1). These include (1) tetrahydrobiopterin (BH_4_), synthesized by the GTP cyclohydrolase 1 (GCH1)–6-pyruvoyl-tetrahydropterin synthase (PTS)–sepiapterin reductase (SPR) metabolic pathway and regenerated by dihydrofolate reductase (DHFR)^11^; (2) 7-dehydrocholesterol, the substrate of 7-dehydrocholesterol reductase (DHCR7)^12^; (3) the reduced form of vitamin E (α-tocopherol)^13,14^; (4) the reduced form of vitamin K^15^; and (5) the reduced form of coenzyme Q_10_ (CoQ_10__)_ (CoQ_10_H_2_, ubiquinol)^16,17^. Among these RTAs, the strategy that has recently attracted the greatest interest, particularly in the context of cancer, is targeting the enzymes that regulate the ubiquinol (CoQ_10_H_2_, reduced) pool to sensitize cancer cells to ferroptosis. Notably, the CoQ_10_ redox system represents a particularly attractive therapeutic node because multiple enzymatic pathways converge to maintain the cellular ubiquinol (CoQ_10_H_2_, reduced) pool. Disrupting this network can simultaneously weaken parallel ferroptosis defense mechanisms, thereby lowering the threshold for lipid peroxidation and ferroptotic cell death in cancer cells.

Before CoQ_10_ emerged as a ferroptosis suppressor, it was primarily viewed as an essential electron carrier in the mitochondrial electron transport chain (ETC), shuttling electrons from complexes I/II to complex III to maintain oxidative phosphorylation^18^. Defects in CoQ_10_ biosynthesis, or impairments in the redox machinery required to maintain CoQ_10_ in its reduced form (CoQ_10_H_2_, ubiquinol), can compromise ETC function. Such deficiencies contribute to mitochondrial disorders, including Leigh syndrome, as well as neurodegenerative diseases such as Parkinson’s and Alzheimer’s disease^19–21^. Recent breakthroughs by the Conrad and Olzmann groups have redefined CoQ_10_ biology by identifying ferroptosis suppressor protein 1 (FSP1) as a potent extramitochondrial CoQ_10_ oxidoreductase. FSP1 reduces ubiquinone (CoQ_10_, oxidized) to ubiquinol (CoQ_10_H₂, reduced) at the plasma membrane and lipid droplets (LDs), thereby trapping lipid radicals^16,17,22^. This work also provided a mechanistic explanation for why CoQ_10_ is also found in cellular membranes. In parallel, dihydroorotate dehydrogenase (DHODH) and sulfide:quinone oxidoreductase (SQOR) were shown to generate ubiquinol (CoQ_10_H_2_, reduced) within mitochondria using dihydroorotate and selenide, respectively^23,24^. Together, these discoveries firmly establish CoQ_10_ oxidoreductases as a critical antiferroptotic defense system. Importantly, this pathway is enzymatically tractable, as the enzymes that control CoQ_10_ reduction are druggable oxidoreductases, making the CoQ_10_ axis a promising pharmacological entry point for ferroptosis-based cancer therapy. In addition, GPX4 inhibitors remain challenging to use in vivo owing to their poor bioavailability, and pharmacological inhibition of GPX4 carries a substantial risk of systemic toxicity, as demonstrated in GPX4 knockout mouse models^25–27^ Thus, modulating the CoQ_10_ redox cycle, either by inhibiting ubiquinol (CoQ_10_H_2_, reduced) formation or altering oxidoreductase activity, has emerged as an alternative therapeutic strategy for ferroptosis-based cancer therapy.

Here, we first summarize the canonical CoQ_10_ biosynthetic pathway and provide a comprehensive overview of both established and emerging CoQ_10_ oxidoreductases, such as FSP1, DHODH, SQOR and additional enzymes, emphasizing their biochemical roles in ferroptosis regulation. Finally, we discuss how targeting CoQ_10_ oxidoreductases may create new opportunities for ferroptosis-based cancer therapies. Finally, we discuss how targeting the CoQ_10_ redox network may provide a clinically actionable strategy to exploit ferroptosis vulnerabilities in cancer.

## Main body

### CoQ_10_ synthesis pathway

**Fig. 2:**
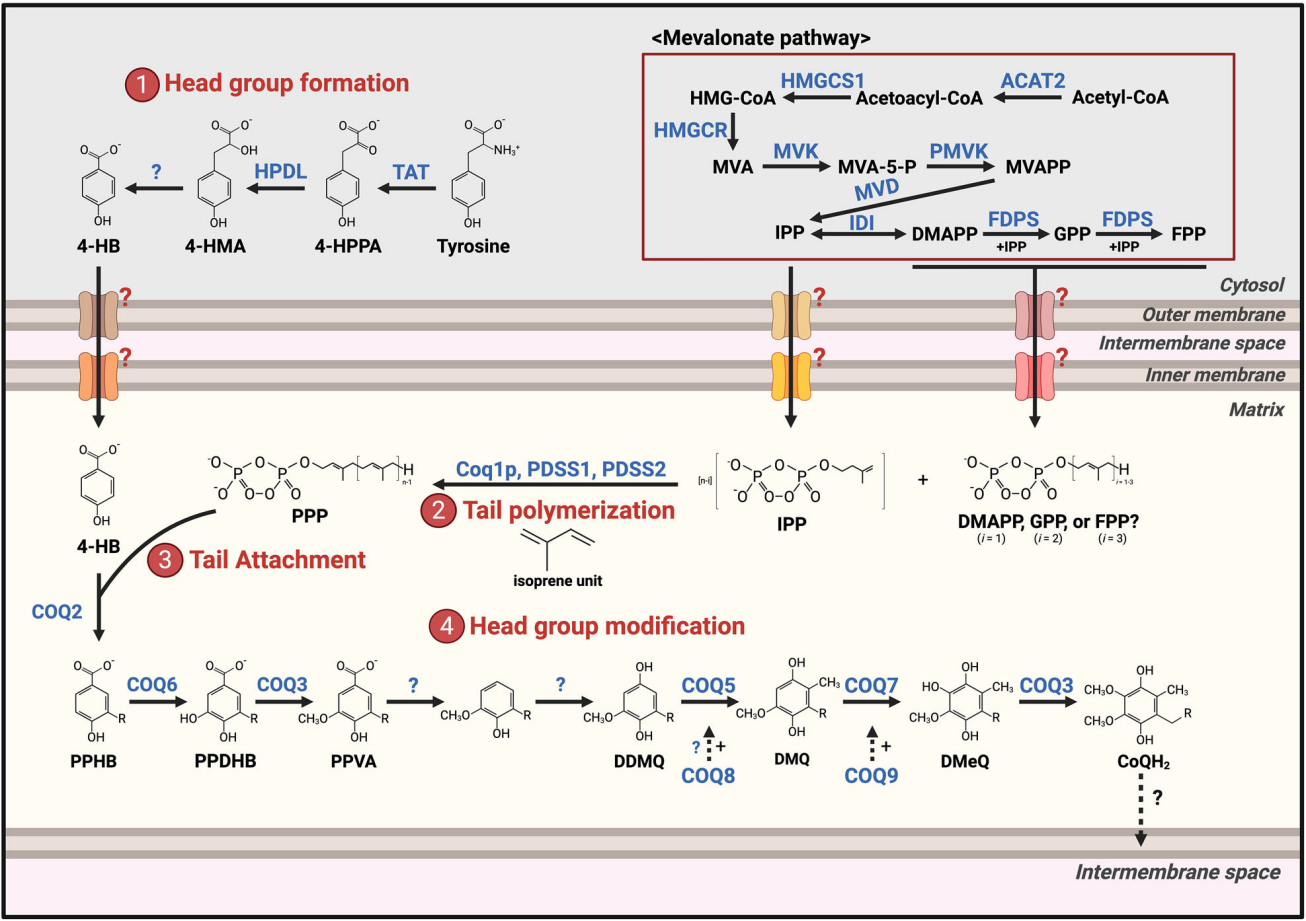
Proposed CoQ_10_ biosynthesis pathway in mammals. This schematic outlines the proposed biosynthetic network for CoQ_10_ in mammalian cells. It integrates cytosolic mevalonate pathway-derived precursors with mitochondrial enzymes responsible for head group prenylation and modification. Several transport steps and enzymatic reactions remain undefined (indicated by question marks), and some mechanistic details are extrapolated from yeast models. Dotted arrows and plus signs denote putative roles of auxiliary proteins COQ8A/B and COQ9. ACAT2, acetyl-CoA acetyltransferase 2; DDMQ, demethoxy-demethyl-coenzyme Q; DMeQ, demethyl-coenzyme Q; DMQ, demethoxy coenzyme Q; FDPS, farnesyl diphosphate synthase; FPP, farnesyl diphosphate; GPP, geranyl pyrophosphate; HMG-CoA, 3-hydroxy-3-methylglutaryl-coenzyme A; HMGCS1, HMG-CoA synthase 1; HMGCR, HMG-CoA reductase; IDI, isopentenyl diphosphate isomerase; MVA, mevalonate; MVA-5-P, mevalonate 5 phosphate; MVAPP, mevalonate pyrophosphate; MVD, mevalonate diphosphate decarboxylase; MVK, mevalonate kinase; PDSS1, prenyl (decaprenyl) diphosphate synthase subunit 1; PMVK, phosphomevalonate kinase; PPDHB, polyprenyl-dihydroxybenzoate; PPVA, polyprenyl vanillic acid.

The biosynthesis of CoQ_10_ involves a series of intricate enzymatic steps^28^. Structurally, CoQ_10_ comprises a long polyisoprenoid lipid tail and a quinone head group, with redox activity localized in the head group. Its biosynthetic pathway can be broadly divided into four stages (Fig. 2): (1) synthesis of the head group, (2) synthesis of the isoprenoid tail, (3) condensation of the tail and head group and (4) postcondensation modification of the head group.

1. Synthesis of the head group: In mammals, the conserved head group precursor for CoQ_10_ is 4-hydroxybenzoate (4-HB), which is derived from tyrosine. Tyrosine is initially converted to 4-hydroxyphenylpyruvate (4-HPPA) via tyrosine aminotransferase (TAT). However, the subsequent enzymatic step, conversion of 4-HPPA to 4-hydroxymandelate (4-HMA), remained unknown for many years^28^. Recently, the Pacold group identified 4-HPPA dioxygenase-like protein (HPDL) as the enzyme responsible for this transformation, using ^18O_2_ gas labeling and metabolomics techniques^29^. In a follow-up study, the same group demonstrated that HPDL deficiency induces a lethal form of mitochondrial encephalopathy and, importantly, supplementation with HPDL-derived intermediates (4-HMA or 4-HB) was sufficient to alleviate disease symptoms. These findings suggest that direct administration of CoQ_10_ head-group intermediates may be a more effective therapeutic strategy than full CoQ_10_ supplementation^30^. Despite these advances, the final step in the pathway—the conversion of 4-HMA to 4-HB—remains unresolved, and the enzyme responsible for this transformation has yet to be identified. Furthermore, for the isoprenoid tail to be attached, 4-HB must be transported into the mitochondrial matrix, where the condensation with the tail occurs. However, the molecular mechanism underlying 4-HB transport into mitochondria remains poorly understood and warrants further investigation.
2. Synthesis of the isoprenoid tail: The isoprenoid tail of CoQ is synthesized through the cytosolic mevalonate pathway, which produces key precursors such as isopentenyl pyrophosphate (IPP) and dimethylallyl pyrophosphate (DMAPP)^28,31^. These isoprenoid units are then transported into the mitochondrial matrix, although the specific transport mechanisms remain unclear. Within the mitochondria, polyprenyl diphosphate synthase complex, composed of PDSS1 and PDSS2, catalyzes the sequential head-to-tail polymerization of these units to form a polyprenyl diphosphate chain of species-specific length. For instance, humans synthesize CoQ_10_ with ten isoprene units, mice produce CoQ_9_, *Escherichia* *coli* synthesizes CoQ_8_, and *Saccharomyces cerevisiae* generates CoQ_6_^28,32,33^. This chain length variability contributes to differences in CoQ function and membrane dynamics across organisms.
3. Condensation of the tail and head group: Once both the benzoquinone head group precursor (4-HB) and the isoprenoid side chain (polyprenyl pyrophosphate, PPP) are synthesized, the enzyme COQ2 catalyzes their condensation via an electrophilic aromatic substitution^33^. This reaction forms polyprenyl-4-hydroxybenzoate (PPHB), the first committed intermediate in CoQ biosynthesis that links the aromatic ring with the hydrophobic tail.
4. Postcondensation modification of the head group: After formation of PPHB, the head group undergoes a series of enzymatic modifications—including decarboxylation, hydroxylation and methylation—to produce the final redox-active molecule, CoQ. These modifications are catalyzed by a conserved set of enzymes: COQ3, COQ5, COQ6, COQ7 and COQ9. Most of our understanding of this multistep pathway stems from studies in yeast and bacteria, where the sequential actions of these enzymes have been genetically and biochemically dissected^28,33^. However, the exact order, timing and coordination of these modifications in mammalian cells remain incompletely defined. In addition, accessory proteins such as COQ4, COQ8A/B, COQ9 and COQ10A/B are believed to facilitate enzyme complex assembly, substrate channeling or stabilization of the biosynthetic complex (commonly referred to as the ‘CoQ synthome’) but their precise molecular functions are still under investigation. In addition to the canonical CoQ biosynthetic enzymes, recent studies have revealed that reticulon 4-interacting protein 1 (RTN4IP1), a mitochondrial matrix protein, participates in CoQ_9_ biosynthesis by regulating the activity of COQ3^34^. This example suggest that, beyond the core enzymatic machinery, additional regulatory factors may modulate the activity of CoQ_9_ biosynthetic enzymes. Further studies will therefore be required to elucidate how such regulatory mechanisms contribute to the control of CoQ_10_ biosynthesis in mammalian cells.

Importantly, elucidating the currently unresolved steps of the CoQ_10_ biosynthetic pathway has direct implications for ferroptosis biology and therapeutic development. As regulation of CoQ_10_ synthesis influences the cellular CoQ_10_ pool, it is expected to affect cellular susceptibility to ferroptosis. For example, recent studies have shown that perturbation of COQ2, a key enzyme responsible for coupling the isoprenoid tail to the quinone head group, increases ferroptosis sensitivity, and thus targeting COQ2 enhances ferroptotic vulnerability in multiple myeloma cells^35^. In addition, proteins involved in CoQ trafficking also contribute to ferroptosis regulation; for instance, STARD7 has been shown to control mitochondrial to plasma membrane CoQ distribution, thereby influencing cellular susceptibility to ferroptotic stress^36^. Consistently, loss of STARD7 increases ferroptosis sensitivity in therapy-resistant colorectal cancer models by reducing the delivery of CoQ to the plasma membrane. Together, these findings suggest that both CoQ biosynthesis and intracellular transport represent potential therapeutic nodes for modulating ferroptosis, particularly in disease contexts such as therapy-resistant cancers.

### Established (canonical) roles of CoQ_10_ oxidoreductases

**Fig. 3:**
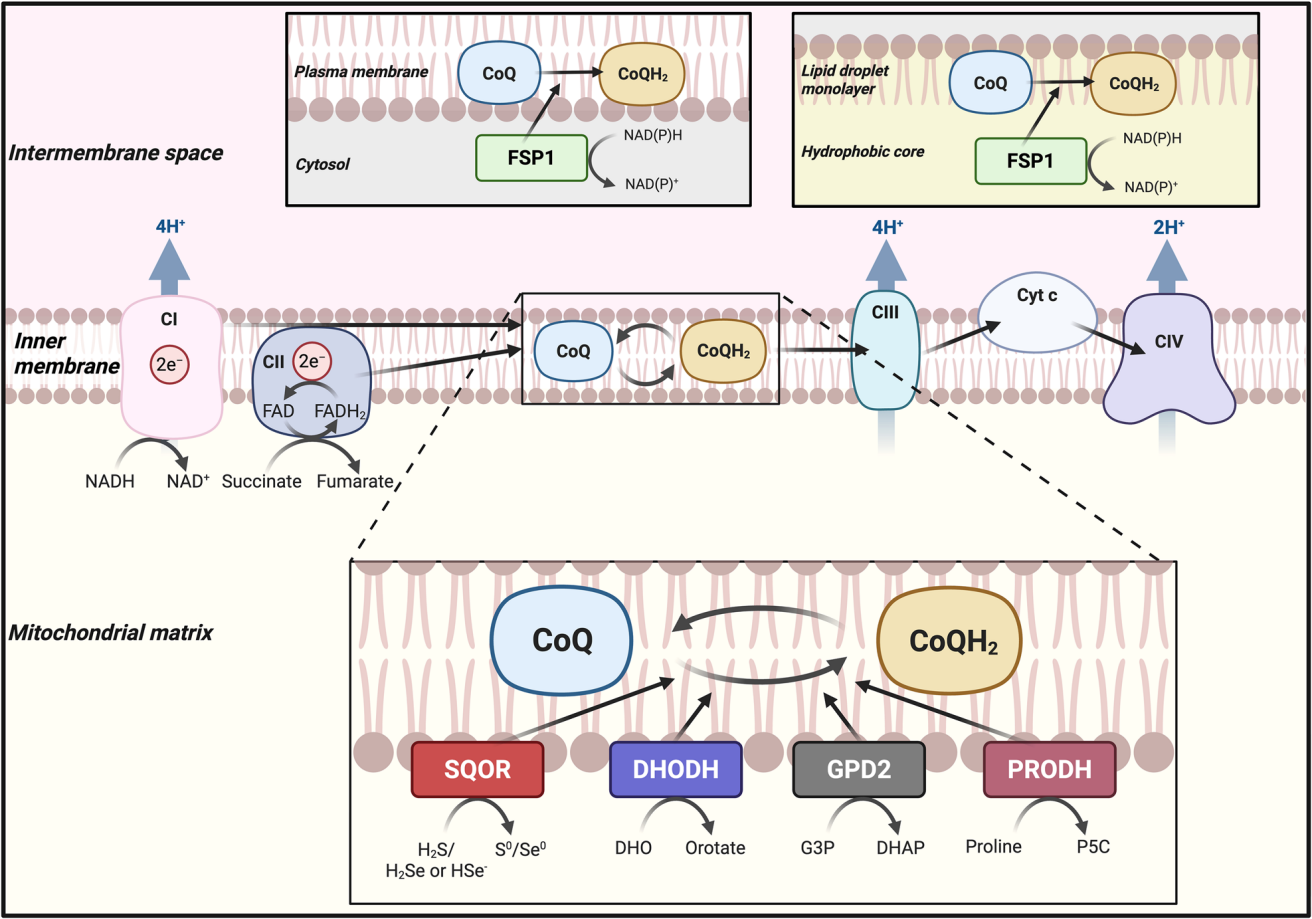
Subcellular localization and enzymatic sources of CoQ_10_ reduction across cellular compartments. This schematic illustrates key CoQ_10_ oxidoreductases and their associated metabolic substrates contributing to the generation of ubiquinol (CoQ_10_H_2_) in distinct cellular compartments. In the plasma membrane (top-left inset), FSP1 utilizes NAD(P)H to reduce CoQ_10_ to CoQ_10_H_2_, counteracting lipid peroxidation and ferroptosis. A similar NAD(P)H-dependent FSP1-mediated reduction occurs at the LD monolayer (top-right inset), protecting neutral lipids such as triglycerides and cholesteryl esters from oxidative damage. Within mitochondria, CoQ_10_ acts as an electron carrier within the ETC. Electrons derived from NADH and FADH_2_ are transferred to CoQ_10_ via complex I (CI) and complex II (CII), respectively, resulting in its reduction to CoQ_10_H_2_, which then shuttles electrons to complex III (CIII), facilitating proton translocation across the inner mitochondrial membrane. Additional inner-membrane oxidoreductases—including SQOR, DHODH, mitochondrial GPD2 and PRODH—contribute to CoQ_10_ reduction by oxidizing distinct substrates (for example, H_2_S, dihydroorotate, G3P and proline) and transferring the resulting electrons to CoQ_10_. Collectively, these pathways enhance mitochondrial bioenergetics and antioxidant capacity via dynamic regulation of CoQ redox state.

Although CoQ_10_ oxidoreductases have recently gained attention for their role in generating ubiquinol (CoQ_10_H_2_), a potent lipid RTA, their long-established function is to serve as electron carriers in the mitochondrial ETC. Specifically, they facilitate electron transfer from complex I,complex II and other mitochondrial oxidoreductases to complex III. Moreover, FSP1, a CoQ_10_ oxidoreductase recently identified at the plasma membrane, was previously known as apoptosis-inducing factor mitochondria-associated 2 (AIFM2)^37^. In this section, we highlight the classical roles of CoQ_10_ oxidoreductases, with a focus on their canonical functions in mitochondrial respiration and redox biology (Fig. 3).

1. FSP1/AIFM2: FSP1 was originally classified as a caspase-independent apoptosis-inducing protein, on the basis of its sequence similarity to apoptosis-inducing factor 1 (AIFM1), and was annotated as a flavoprotein with NAD(P)H oxidase activity^38^. Several early studies implicated AIFM2 in the regulation of apoptosis. For instance, one report proposed a model in which AIFM2 attenuates survival signaling in response to cytosolic foreign DNA, thereby contributing to apoptotic cell death^37,39^. Another study suggested that oxidative stress-induced 4-hydroxy-2-nonenal (HNE) triggers mitochondrial translocation of AIFM2, promoting apoptosis in cardiac tissues of both mice and humans^40^. In lung cancer models, AIFM2 overexpression was reported to enhance apoptosis in response to cytotoxic drug treatment^39^. However, conflicting findings have also emerged. In murine erythroleukemia (MEL) cells, AIFM2 did not affect apoptotic status but instead promoted erythroid differentiation^41^. Moreover, many of the early studies linking AIFM2 to apoptosis relied on predictive models, lacked apoptosis-specific markers or failed to demonstrate functional rescue using apoptosis inhibitors. Consequently, the canonical role of AIFM2 in apoptosis remained inconclusive until more recent discoveries redefined its function as a key suppressor of ferroptosis.
2. DHODH: DHODH is a mitochondrial flavoprotein localized to the inner mitochondrial membrane, where it catalyzes the fourth and rate-limiting step of de novo pyrimidine biosynthesis—the oxidation of dihydroorotate to orotate^42,43^. This reaction is coupled to the mitochondrial ETC via the reduction of ubiquinone (CoQ_10_) to ubiquinol (CoQ_10_H_2_), linking nucleotide synthesis to mitochondrial respiratory activity. DHODH activity is essential for proliferating cells owing to their increased demand for nucleotides, and DHODH inhibition has long been studied as a therapeutic strategy in autoimmune diseases (for example, via leflunomide) and cancer, where it can lead to cell cycle arrest, DNA damage and replication stress^42,44,45^. These canonical functions positioned DHODH primarily as a metabolic enzyme critical for nucleotide biosynthesis and cellular proliferation, before its more recent identification as a mitochondria-specific ferroptosis suppressor in select cancer contexts.
3. SQOR: SQOR is a mitochondrial inner-membrane flavoprotein that plays a central role in the detoxification of hydrogen sulfide (H_2_S), a gaseous signaling molecule that becomes toxic at high concentrations. SQOR catalyzes the first and rate-limiting step in mitochondrial H_2_S oxidation by transferring electrons from sulfide to ubiquinone (CoQ_10_), forming ubiquinol (CoQ_10_H_2_) and a persulfide intermediate, which is further metabolized by downstream enzymes in the sulfide oxidation pathway^46,47^. This process contributes to cellular redox balance, energy production and sulfide homeostasis, particularly in tissues with high metabolic activity such as the brain, liver and heart^48–51^. Beyond its role in H_2_S detoxification, SQOR is involved in ETC coupling, indirectly supporting ATP synthesis under physiological conditions. Dysregulation of SQOR has been implicated in mitochondrial diseases^52^, neurodegenerative disorders^53^ and ischemia–reperfusion injury^48^, highlighting its broader importance in mitochondrial redox metabolism. These canonical functions defined SQOR as a redox enzyme primarily dedicated to sulfide clearance and CoQ redox cycling, before its emerging role as a ferroptosis suppressor under specific metabolic stresses.
4. mGPDH: Mitochondrial glycerol-3-phosphate (G3P) dehydrogenase (mGPDH, also known as GPD2) is a flavoprotein located in the inner mitochondrial membrane and serves as a key enzyme in the glycerol phosphate shuttle^54^. It catalyzes the oxidation of G3P to dihydroxyacetone phosphate (DHAP), transferring electrons to ubiquinone (CoQ_10_) to form ubiquinol (CoQ_10_H_2_), thereby linking cytosolic NADH to the mitochondrial ETC. This activity supports cytosolic redox balance, particularly under high glycolytic flux, and contributes to energy metabolism and reactive oxygen species (ROS) generation in metabolically active tissues^55^. mGPDH has also been implicated in diseases such as type 2 diabetes and nonalcoholic fatty liver disease^56–58^. These canonical functions positioned mGPDH as a redox-linked metabolic enzyme long before its recent recognition as a ferroptosis regulator via ubiquinol (CoQ_10_H_2_)-dependent antioxidant defense.
5. PRODH: Proline dehydrogenase (PRODH) is a mitochondrial flavoenzyme that catalyzes the first step of proline catabolism, converting proline into Δ^1^-pyrroline-5-carboxylate (P5C) while transferring electrons to ubiquinone (CoQ_10_) in the mitochondrial ETC. Through this reaction, PRODH contributes to both ATP production and mitochondrial ROS generation^59,60^. PRODH is transcriptionally regulated by p53 and plays important roles in apoptosis, autophagy and cellular stress adaptation, particularly under nutrient deprivation or oxidative stress^61,62^. In various cancer models, PRODH has been described as a context-dependent tumor suppressor or promoter, depending on the metabolic state and microenvironment of the cell^63^. A recent study further demonstrated that proline catabolism via PRODH supports 3D growth of breast cancer cells, with elevated PRODH expression and proline oxidation in metastatic lesions compared with primary tumors, thereby promoting metastatic colonization and identifying PRODH as a potential target for metastasis suppression^64^. Beyond cancer, PRODH dysfunction is also linked to neuropsychiatric and metabolic diseases, including schizophrenia^65,66^, and hyperprolinemia type I^67^, reflecting its broader role in regulating mitochondrial redox balance and proline–glutamate neurotransmitter metabolism.
6. Complex I: Complex I (NADH:ubiquinone oxidoreductase) is a large multiprotein complex located in the mitochondrial inner membrane that accepts electrons from NADH and transfers them to ubiquinone (CoQ_10_) via FMN and a series of iron–sulfur (Fe–S) clusters, reducing it to ubiquinol (CoQ_10_H_2_)^68,69^. In addition to catalyzing this major CoQ_10_-reducing reaction, complex I pumps protons into the intermembrane space, generating a proton motive force that drives ATP synthesis^68,70^. As NADH originates from diverse metabolic pathways—including the tricarboxylic acid (TCA) cycle, β-oxidation and amino-acid catabolism—complex I acts as a metabolic control point that couples cellular metabolic flux to electron transport. Consequently, its activity reflects the cell’s energy state and redox balance and can modulate mitochondrial ROS production under conditions of high NADH/NAD^+^ ratios or impaired electron flow. Defects in complex I are among the most common causes of mitochondrial diseases, contributing to disorders such as Leigh syndrome, mitochondrial encephalomyopathy and Parkinson’s disease by disrupting ATP production and elevating oxidative stress^21,71–73^.
7. Complex II: Complex II (succinate dehydrogenase, SDH) is an FAD-dependent enzyme embedded in the mitochondrial inner membrane that oxidizes succinate to fumarate as part of the TCA cycle while transferring the resulting electrons through FAD and a series of iron–sulfur clusters to ubiquinone (CoQ_10_), reducing it to ubiquinol (CoQ_10_H_2_)^74^. As the only enzyme shared between the TCA cycle and the ETC, complex II functions as a key metabolic node that directly feeds carbon and reducing equivalents into mitochondrial respiration and CoQ_10_ redox cycling^75,76^. Notably, under hypoxic or electron transport–limited conditions, SDH can reverse its activity to reduce fumarate to succinate using electrons from the CoQ_10_ pool, a metabolic rerouting, allowing cells to maintain redox balance when oxygen is scarce^77^. Defects in SDH subunits lead to succinate accumulation and oncometabolite-driven pseudohypoxia and are strongly linked to hereditary paragangliomas, pheochromocytomas and certain renal cell carcinomas, underscoring the essential role of complex II in metabolic and redox homeostasis^78,79^.

### CoQ_10_ oxidoreductases in the regulation of ferroptosis

1. FSP1 in the regulation of ferroptosis
  “FSP1 as a therapeutic target in cancer”
  FSP1 (formerly AIFM2) was first identified as a ferroptosis regulator among CoQ_10_ oxidoreductases through independent unbiased genetic screens conducted by the Conrad and Olzmann groups under conditions of GPX4 inhibition^16,17^. Both studies demonstrated that FSP1 acts as an NADH/NADPH-dependent CoQ_10_ reductase that predominantly localizes to the plasma membrane via N-terminal myristoylation—a posttranslational modification essential for its antiferroptotic activity. FSP1 catalyzes the reduction of ubiquinone (CoQ_10_) to ubiquinol (CoQ_10_H_2_), a lipophilic RTA that effectively halts lipid peroxidation chain reactions. This mechanism functions independently of the canonical GPX4–GSH axis, offering a parallel ferroptosis-suppressive pathway. FSP1 expression correlates strongly with ferroptosis resistance across diverse cancer cell lines. Importantly, recent in vivo studies have shown that genetic inhibition of FSP1 alone is sufficient to induce ferroptosis and suppress tumor growth, even in the absence of GPX4 inhibition^80,81^. This has been demonstrated in lung cancer mouse models, chromophobe renal cell carcinoma and colorectal cancer. These findings underscore FSP1’s independent role in tumor maintenance and its potential as a stand-alone therapeutic target.
  “FSP1-mediated reduction of vitamin K—expanding substrate scope”
  Although CoQ_10_ is the primary known substrate of FSP1, emerging evidence indicates that FSP1 exhibits promiscuous substrate specificity. Notably, FSP1 can also reduce vitamin K and its derivatives, which share a quinone backbone similar to that of CoQ_10_^15,82^. Reduced forms of vitamin K have RTA properties, suggesting that FSP1 not only supports CoQ_10_ recycling but also expands the intracellular antioxidant pool by processing other quinone-like molecules. This enzymatic versatility highlights FSP1’s broader role in redox regulation beyond CoQ_10_ metabolism. It also raises the possibility that FSP1 inhibitors may disrupt multiple parallel antioxidant systems, potentially enhancing therapeutic efficacy in oxidative stress-dependent pathologies, including cancer and degenerative diseases.
  “FSP1 at lipid droplets—a new physiological frontier”
  More recent studies have revealed that FSP1 is not restricted to the plasma membrane but can also localize to LDs, where it plays a key role in suppressing lipid peroxidation within stored neutral lipids such as triglycerides and cholesteryl esters. In vitro and cellular assays demonstrated that FSP1 prevents polyunsaturated fatty acid (PUFA)-enriched neutral lipid oxidation in LDs, thereby protecting cells from LD-mediated ferroptosis^22^. Cells deficient in FSP1 accumulate oxidized lipids within LDs, and PUFA supplementation further sensitizes these cells to ferroptotic death. These findings expand the role of FSP1 beyond membrane defense, suggesting that it serves as a general lipid quality control enzyme across cellular compartments. Importantly, this implies that FSP1 may be essential not only in cancer resistance to ferroptosis but also in physiological contexts such as adipose tissue metabolism, where regulation of neutral lipid oxidation is critical for maintaining tissue homeostasis.
2. DHODH in the regulation of ferroptosis
  “Which organelle membranes initiate and propagate ferroptosis?”
  One of the central questions in ferroptosis research concerns which subcellular membranes serve as the critical sites of lipid peroxidation that initiate this unique, iron-dependent form of cell death. Early studies pointed to the plasma membrane as the primary execution site, supported by findings that FSP1, as described above, exerts its antiferroptotic function through the reduction of plasma membrane ubiquinone (CoQ_10_)^16,17^. However, growing evidence has since expanded this view, implicating intracellular organelles such as the endoplasmic reticulum (ER)^83,84^, mitochondria^23,85–87^, peroxisomes^88^ and lysosomes^89^ could be contributors. Among these, the ER has emerged as a central hub for initiating lipid peroxidation, supported by imaging and lipidomics data showing that oxidation events in the ER precede those at the plasma membrane^83^. Mitochondria, rich in polyunsaturated phospholipids and ROS, have also been highlighted, particularly under metabolic stress^23^. In addition, membrane contact sites, such as ER–mitochondria and ER–lysosome interfaces, are being actively investigated as potential conduits for lipid peroxidation signals^90,91^. Together, these findings reflect a paradigm shift from a static, single-organelle model to a dynamic, organelle-interactive framework of ferroptosis, with context-dependent roles for each membrane system.
  In this section, we focus on DHODH and other CoQ_10_ oxidoreductases that reduce mitochondrial CoQ_10_ to its antioxidant form, ubiquinol (CoQ_10_H_2_). CoQ_10_ is synthesized in the inner mitochondrial membrane, but can subsequently traffic to other membrane systems, including the ER and plasma membrane, via vesicular transport or organelle contact sites^28,36^. This interorganelle distribution suggests that mitochondrial CoQ_10_ oxidoreductases, such as DHODH, may influence ferroptosis beyond mitochondria by maintaining the redox state of CoQ_10_ pools across multiple cellular membranes. Thus, understanding how CoQ_10_ is mobilized and utilized across organelles is crucial for decoding the spatial regulation of ferroptosis.
  An important conceptual implication of CoQ_10_ biology is that, although CoQ_10_ is synthesized in mitochondria, its protective function extends to multiple membrane compartments throughout the cell. Instead of acting solely within mitochondria, CoQ_10_ can be distributed to membranes such as the ER, plasma membrane and LDs, where its reduced form, ubiquinol, suppresses lipid peroxidation^17,22,36^. This spatial organization suggests that the site of ferroptosis initiation may depend not only on where lipid peroxidation first arises but also on where CoQ_10_ is available and efficiently maintained in its reduced state. In this framework, mitochondrial defects in CoQ_10_ synthesis or redox cycling could influence ferroptosis susceptibility beyond mitochondria by weakening antioxidant protection across interconnected membrane compartments. Conversely, impaired CoQ_10_ trafficking or compartment-specific depletion of ubiquinol may allow lipid peroxidation to originate in one organelle and propagate to others, potentially through membrane contact sites. Thus, the intracellular distribution and compartment-specific regeneration of CoQ_10_ may be key determinants of both the membrane origin and the spatial progression of ferroptosis.
  “Controversy over the role of DHODH in ferroptosis regulation”
  Although ferroptosis was initially thought to be primarily executed at the plasma membrane, a pivotal study by Boyi Guan’s group highlighted mitochondria as a potential site of critical lipid peroxidation, particularly under conditions of GPX4 inactivation or in GPX4-low cell lines^23^. In this context, DHODH, a mitochondrial inner-membrane enzyme traditionally known for its role in de novo pyrimidine biosynthesis, was proposed to also function as a CoQ_10_ oxidoreductase and ferroptosis suppressor. DHODH reduces ubiquinone (CoQ_10_) to ubiquinol (CoQ_10_H_2_), thereby mitigating mitochondrial lipid peroxidation and delaying ferroptosis in GPX4-deficient settings. Notably, the DHODH inhibitor brequinar was shown to suppress tumor growth in vivo, an effect partially attributed to enhanced ferroptotic vulnerability in GPX4-low tumors. These findings positioned DHODH as a third axis of ferroptosis defense, alongside GPX4 and FSP1, particularly in mitochondria-rich or metabolically active cell types.
  However, this hypothesis has been challenged. A 2023 study by Mishima et al. reported that genetic deletion or pharmacological inhibition of DHODH did not substantially alter ferroptosis sensitivity across multiple cellular models, suggesting that its protective role may be overstated or highly context dependent^92^. They also showed that the DHODH inhibitor brequinar used actually inhibits FSP1 activity. Nevertheless, several follow-up studies have reaffirmed DHODH’s ferroptosis‑suppressive function under specific metabolic or oxidative‑stress conditions^93–96^, supporting the notion of a context‑dependent mechanism rather than a universally essential role.
3. SQOR in the regulation of ferroptosis
  “SQOR utilizes both hydrogen sulfide and hydrogen selenide to reduce ubiquinone (CoQ_10_) to ubiquinol (CoQ_10_ H_2_)”
  Recent studies have expanded the list of cellular enzymes capable of reducing CoQ_10_ and modulating ferroptosis, with SQOR emerging as a notable addition alongside FSP1 and DHODH. Similar to FSP1, SQOR exhibits promiscuous enzymatic activity, capable of processing structurally similar substrates^24,47^. While traditionally recognized for its role in hydrogen sulfide oxidation within mitochondria, SQOR has now been shown to also utilize hydrogen selenide as an alternative electron donor for the reduction of ubiquinone (CoQ_10_) to ubiquinol (CoQ_10_H_2_)^24^. This discovery leverages the chemical similarity between sulfur and selenium and uncovers a previously unappreciated role for selenide, a selenium metabolic intermediate. Beyond its established function in selenocysteine synthesis for the production of selenoproteins such as GPX4, selenide can now be viewed as an active participant in mitochondrial antioxidant defense. Notably, SQOR deficiency leads to reduced cellular ubiquinol (CoQ_10_H_2_) levels and increased sensitivity to ferroptotic stress, positioning SQOR as another mitochondria-resident suppressor of ferroptosis.
  “SQOR-mediated persulfide formation, another anti-ferroptotic mechanism”
  Beyond its role in ubiquinol (CoQ_10_H_2_) generation, SQOR also contributes to ferroptosis resistance through the production of persulfide-containing metabolites. During the oxidation of hydrogen sulfide, SQOR generates elemental sulfur (S^0^), which can react with intracellular thiols, such as GSH, to form GSH persulfide (GSSH) or thiosulfate^97^. These hydropersulfides have been identified as potent radical scavengers capable of suppressing lipid peroxidation, a key step in ferroptosis execution^97^. Experimental evidence from models of acute kidney injury supports this protective function of SQOR: through persulfide production^98^. Collectively, these findings establish SQOR as a multifunctional regulator of ferroptosis, linking sulfur and selenium metabolism to redox homeostasis and membrane integrity.
4. mGPDH in the regulation of ferroptosis
  “G3P metabolism supports ferroptosis defense in disease contexts”
  In the first study identifying mGPDH as a ferroptosis regulator, metabolomic analyses revealed that treatment of cancer cells with GPX4 inhibitors led to a marked depletion of intracellular glycerol‑3‑phosphate (G3P)^99^. This finding prompted further investigation into the role of G3P‑utilizing mGPDH in ferroptosis defense. mGPDH catalyzes the oxidation of G3P while reducing ubiquinone (CoQ_10_) to ubiquinol (CoQ_10_H_2_), thereby helping to sustain the mitochondrial CoQ_10_H_2_ pool and suppress lipid peroxidation. Loss of mGPDH sensitized cells to GPX4 inhibitor-induced ferroptosis, whereas supplementation with G3P rescued this vulnerability in a mGPDH-dependent manner. More recently, mGPDH’s protective function was validated in vivo: in a mouse model of sepsis-induced acute lung injury, mGPDH knockdown exacerbated lung injury, vascular leakage, inflammation and oxidative damage, accompanied by elevated ferroptotic markers^100^. Activation of GPX4 partially rescued these effects, reinforcing the role of mGPDH as an auxiliary mitochondrial defense mechanism against ferroptosis under pathological stress.
5. PRODH in the regulation of ferroptosis
  “Potential links between proline metabolism and ferroptosis”
  PRODH is a mitochondrial enzyme primarily responsible for the oxidation of proline to P5C as part of amino acid catabolism and redox metabolism^101^. Recent insights suggest that this pathway may also play a role in ferroptosis regulation. During proline catabolism, electrons derived from PRODH activity are transferred to the mitochondrial ETC, potentially supporting the regeneration of ubiquinol (CoQ_10_H_2_). In this regard, PRODH may act in a manner analogous to other mitochondrial CoQ_10_ oxidoreductases, helping to maintain the redox state of CoQ_10_ under conditions of oxidative stress. Although experimental validation of PRODH’s role in ferroptosis remains limited across various model systems, a recent study demonstrated that PRODH suppresses tamoxifen-induced ferroptosis in breast cancer cell lines, suggesting a context-dependent antiferroptotic function^102^. These findings support the notion that mitochondrial amino acid metabolic pathways, particularly those involving redox-active substrates such as proline, may serve as auxiliary regulators of ferroptosis, operating alongside canonical CoQ_10_-handling enzymes such as FSP1 or SQOR.
6. Complex I in the regulation of ferroptosis
  “Complex I: a context-dependent regulator of ferroptosis”
  Complex I (NADH:ubiquinone oxidoreductase), the primary entry point of electrons into the mitochondrial ETC, has been implicated in ferroptosis regulation through its involvement in maintaining the redox state of CoQ_10_. Multiple studies have demonstrated that both pharmacological inhibition and genetic ablation of complex I can influence ferroptosis sensitivity in various disease contexts, including cancer, skin photoaging, Parkinson’s disease and intracerebral hemorrhage^103–106^. Mechanistically, inhibition of complex I is expected to reduce mitochondrial ubiquinol (CoQ_10_H_2_) levels, promoting lipid peroxidation and sensitizing cells to ferroptosis^107^. However, this relationship is not straightforward and appears to be context dependent. For instance, pharmacological inhibitors of complex I not only deplete CoQ_10_H_2_ but also impair ATP production, which activates AMP-activated protein kinase (AMPK)^108^. AMPK activation, in turn, suppresses ferroptosis by inhibiting PUFA biosynthesis through phosphorylation of acetyl-CoA carboxylase^109^. Therefore, complex I inhibition can exert both pro- and antiferroptotic effects: promoting ferroptosis via ubiquinol (CoQ_10_H_2_) depletion, while simultaneously suppressing it through AMPK activation. This duality is particularly evident in LKB1-deficient cancers, where AMPK is inactive. In such settings, complex I inhibition fails to activate AMPK, resulting in enhanced ferroptosis sensitivity and synergistic antitumor effects when combined with radiotherapy^108^. By contrast, genetic ablation of complex I, which does not trigger AMPK activation, uniformly increases ferroptosis susceptibility by eliminating the compensatory antiferroptotic pathway. In addition, recent studies suggest that under certain conditions, such as hyperreduced CoQ pools and elevated mitochondrial membrane potential, reverse electron transport may occur—whereby electrons flow from ubiquinol (CoQ_10_H_2_) back to complex I, generating NADH^110,111^. Reverse electron transport is also associated with ROS generation at complex I. The role of complex I in ferroptosis under such ‘reverse’ ETC conditions remains unexplored but may diverge markedly from its function in forward ETC. Together, these findings highlight the complex and context-specific role of complex I in ferroptosis regulation. The interplay between CoQ redox balance, AMPK activation and ETC directionality suggests that the ferroptotic outcome of complex I inhibition depends not only on its biochemical activity but also on the metabolic state of the cell. Future research is needed to clarify how ETC dynamics shape ferroptosis sensitivity across diverse pathophysiological contexts.
7. Complex II (SDH) in the regulation of ferroptosis
  “Unexpected pro-ferroptotic potential of complex II”
  Complex II, also known as SDH, has been extensively studied for its role in the ETC. However, its involvement in ferroptosis has received relatively little attention. Given that SDH oxidizes succinate to fumarate while reducing CoQ_10_, one might predict that it functions similarly to other mitochondrial CoQ_10_ oxidoreductases in suppressing ferroptosis by promoting ubiquinol (CoQ_10_H_2_) production. Contrary to this expectation, recent studies have revealed an unexpected proferroptotic role for complex II. For example, in the context of clear cell renal cell carcinoma development, loss of SDH genes has been shown to dampen oxidative phosphorylation and reduce susceptibility to ferroptosis^112^. Another study reported that SDH activity increases ROS accumulation and promotes lipid peroxidation, thereby facilitating ferroptotic cell death^113^. In addition, inhibition of SDH was found to decrease RSL3-induced ferroptosis, suggesting that SDH may play a role in promoting ferroptosis. Although the precise molecular mechanisms remain unclear, it has been proposed that complex II inhibition helps to maintain mitochondrial redox balance and supports mitochondrial energy metabolism. Taken together, these findings suggest that—contrary to initial assumptions—complex II may contribute to ferroptosis not by protecting cells via ubiquinol (CoQ_10_H_2_) production but by promoting mitochondrial ROS accumulation. Further studies are warranted to elucidate the dual role of complex II in regulating redox balance and ferroptosis, particularly across different cellular and metabolic contexts.
8. CoQ_10_ synthesis and cellular distribution in the regulation of ferroptosis
  “CoQ2 and STARD7: key regulators and therapeutic targets in ferroptosis modulation”
  Cellular resistance to ferroptosis may depend not only on maintaining a robust pool of ubiquinol (CoQ_10_H_2_) but also on the overall size of the cellular CoQ_10_ pool. CoQ_10_ biosynthesis is a multienzyme process culminating in the prenylation of the benzoquinone head group (for example, by COQ2) and subsequent modifications by the CoQ synthesis enzymes as shown in Fig. 1. Defects in CoQ biosynthetic enzymes have long been linked to neurodegenerative diseases, nephropathy and other symptoms related to oxidative stress, neuroinflammation and cellular degeneration^114^. Experimental evidence supports the functional importance of CoQ biosynthesis in ferroptosis suppression: recent unbiased screens in myeloma cells identified COQ2 as a factor promoting proliferation and suppressing lipid peroxidation, suggesting enhanced resistance to ferroptosis^35^. Moreover, in models of ischemia–reperfusion injury and in KEAP1-inactivated lung cancer, inhibition or genetic ablation of CoQ biosynthetic enzymes restored sensitivity to ferroptosis or radiotherapy^115,116^, underscoring the reliance of some cancer cells on an abundant CoQ pool for redox defense and survival.
  Beyond synthesis, proper intracellular transport and distribution of CoQ after mitochondrial production is critical. The lipid‑transfer protein STARD7 has recently been shown to mediate the export of CoQ from mitochondria to other cellular membranes, including the plasma membrane; loss of STARD7 disrupts this transport and increases ferroptosis sensitivity^36^. These findings highlight that efficient CoQ trafficking, in conjunction with robust biosynthesis, constitutes a coordinated ‘CoQ homeostasis network’ essential for ferroptosis defense across multiple membranes.

### Implication of CoQ_10_ oxidoreductases in cancer therapy

The recent recognition of CoQ_10_ oxidoreductases as actionable ferroptosis gatekeepers has opened new opportunities for cancer therapy. Unlike direct GPX4 inhibition, which remains difficult to deploy in vivo owing to bioavailability and systemic toxicity concerns, targeting enzymes that sustain the reduced CoQ_10_ (CoQ_10_H_2_, ubiquinol) pool may provide a more tractable and context-selective strategy to lower the ferroptotic threshold in tumors. In particular, a growing body of in vivo evidence supports the concept that some cancers become functionally dependent on FSP1, creating a therapeutic vulnerability that can be exploited either as monotherapy in specific contexts or as combination therapy with ferroptosis-inducing treatments.

#### FSP1 as an emerging cancer dependency and therapeutic target

FSP1 has progressed from a GPX4-parallel ‘backup’ system to a tumor-maintenance factor in multiple in vivo settings. A key conceptual advance is that FSP1 dependency can be microenvironment driven. A recent study demonstrated that melanoma cells in lymph node metastases shift toward FSP1 dependence, consistent with altered ferroptosis defense requirements in the lymph node niche; importantly, suppressing this axis reduced metastatic fitness, supporting FSP1 as a context-selective vulnerability in melanoma progression^117^. Beyond melanoma, lung adenocarcinoma has emerged as a compelling indication for FSP1-targeted approaches. Pharmacologic inhibition of FSP1 provided notable therapeutic benefit in preclinical lung cancer models, and FSP1 expression had prognostic associations in lung cancer, supporting translational relevance^80^. Consistent with this expanding in vivo landscape, additional tumor contexts are now being reported where FSP1 inhibition alone can restrict tumor growth, including chromophobe renal cell carcinoma, where FSP1 inhibition decreased tumor growth substantially in vivo and synergized with GPX4/SLC7A11 perturbation in vitro^81^. Together, these studies argue that FSP1 can represent (1) a context-dependent essentiality (for example, metastatic niches) and/or (2) a stand-alone oxidative defense node that becomes rate limiting for tumor survival under physiological stressors encountered in vivo.

#### Therapeutic modalities of FSP1 inhibitors and rational combinations

Multiple chemical strategies have been developed to inhibit FSP1. FSEN1 was identified as a small-molecule inhibitor of FSP1 through chemical screening^118^, and subsequent structural analyses revealed that FSEN1 occupies the substrate-binding pocket of FSP1 in a position analogous to CoQ observed in the enzyme active site, providing structural insight into inhibitor recognition^119^. Structural characterization further demonstrated that FSEN1 interacts with a critical phenylalanine residue that is absent in mouse FSP1, explaining the human-specific activity of FSEN1. In addition, iFSP1 was shown to inhibit FSP1 in a species-dependent manner, revealing structural features that confer selectivity for the human enzyme^120^. Another study further demonstrated that the FAD/NAD(P)H-binding site of FSP1 is evolutionarily conserved among NADH quinone reductases, and targeting this region enabled the development of viFSP1, a species-independent inhibitor that interacts with the NAD(P)H-binding pocket of FSP1^121^. Mechanistically distinct compounds have also been reported, including icFSP1, which targets FSP1 through a phase separation-linked mechanism and can synergize with ferroptosis-inducing agents^122^. Collectively, these studies highlight multiple pharmacological strategies to disrupt FSP1 function, including active-site inhibition, species-selective targeting and structure-guided inhibitor development. These reagents support two clinically relevant concepts:

1. Combination sensitization: FSP1 blockade can sensitize tumors to upstream ferroptosis-inducing nodes (for example, GPX4 inhibition or cystine import disruption), consistent with the idea that tumors rely on redundant antioxidant systems. This combination logic is particularly relevant in therapy-resistant states where ferroptosis defense is upregulated.
2. Microenvironment-informed targeting: The melanoma lymph node work suggests that in vitro drug response may underestimate efficacy because FSP1 dependence can be imposed by in vivo constraints^117^.

Notably, nanomedicine or delivery-based approaches are also being explored to co-administer ferroptosis-inducing drugs with FSP1 inhibitors, highlighting a practical path for improving tumor-selective exposure and therapeutic index in vivo^123^.

#### Beyond FSP1 there are therapeutic opportunities across the CoQ_10_ oxidoreductase network

While FSP1 currently has the strongest ‘targetability’ signal, other CoQ_10_ oxidoreductases may be therapeutically leveraged in defined genetic, metabolic or tissue contexts:

1. DHODH axis (mitochondrial defense in select contexts): DHODH connects pyrimidine synthesis to mitochondrial CoQ reduction, and in some settings, DHODH inhibition has been proposed to heighten ferroptosis sensitivity, especially in GPX4-low states. However, DHODH’s role appears context dependent and debated, suggesting that patient stratification (for example, mitochondrial redox burden, GPX4/FSP1 status and metabolic state) will probably determine where DHODH inhibition provides ferroptosis-relevant benefit.
2. SQOR axis (sulfur/selenium metabolism-linked redox defense): SQOR can feed electrons into the CoQ pool and also generate reactive sulfur species (persulfides) with antioxidant potential, positioning it as a multifunctional regulator of lipid peroxidation defense. Therapeutically, this raises two complementary possibilities: (1) SQOR-high tumors may exhibit enhanced mitochondrial ferroptosis resistance through maintained CoQ_10_H_2_ and sulfur-based RTAs and (2) tumors with altered sulfur/selenium metabolism may reveal synthetic vulnerabilities when SQOR-linked defense is impaired. In practice, these hypotheses will probably require metabolic stratification and careful management of systemic sulfur/selenium physiology.
3. Metabolic shuttles and ETC nodes (mGPDH, complex I/II and PRODH): These enzymes can influence the CoQ redox state indirectly through substrate oxidation and electron flux. From a therapeutic standpoint, their main opportunity may be combination design: pairing ferroptosis induction with interventions that remodel mitochondrial electron flow or substrate availability, thereby constraining the capacity to regenerate CoQ_10_H_2_. Given the known pleiotropy of ETC inhibition (for example, AMPK activation and ATP depletion), these approaches will probably require context-aware deployment rather than broad application.

#### Outlook toward biomarker-driven ferroptosis therapies

A central challenge and opportunity will be to define which tumors are truly CoQ–oxidoreductase dependent in vivo. Emerging evidence indicates that dependence can be shaped by metastatic niche, antioxidant nutrient availability and tumor genotype (for example, NRF2/KEAP1 signaling, baseline GPX4 expression and lipid composition). Therefore, future clinical translation will probably benefit from biomarker-guided frameworks, incorporating (1) FSP1 abundance/localization, (2) CoQ pool size and redox state, (3) lipid peroxidation signatures and (4) metabolic context (mitochondrial activity and sulfur/selenium flux). These parameters may be evaluated using tumor biopsy specimens and patient-derived samples through a combination of complementary experimental and clinical methodologies, including immunohistochemistry or spatial proteomics to assess FSP1 expression and localization, metabolomics or lipidomics-based omics approaches to quantify CoQ redox state and lipid peroxidation signatures and functional ferroptosis-sensitivity assays in patient-derived tumor models such as organoids or xenografts. As ferroptosis moves toward therapeutic reality, CoQ_10_ oxidoreductases, particularly FSP1, stand out as a promising class of targets that may enable tumor-selective weakening of antioxidant defenses to unlock ferroptosis-based cancer treatment.

## Closing remarks and future perspectives

Collectively, recent discoveries have repositioned CoQ_10_ from a ‘housekeeping’ electron carrier to a dynamic, compartmentalized redox hub that actively determines ferroptosis sensitivity. The emerging picture is that ferroptosis is not governed by a single antioxidant axis but rather by an interconnected network of radical-trapping metabolites and enzymatic recycling systems. Within this network, CoQ_10_ oxidoreductases occupy a privileged position: they couple substrate availability and electron flow to membrane protection by controlling the abundance of ubiquinol (CoQ_10_H_2_). This framework provides a mechanistic explanation for why tumors—particularly those operating near the limits of redox tolerance—can become dependent on CoQ_10_ redox cycling as a ‘last-mile’ defense against lipid peroxidation. Consequently, disruption of this redox buffering system may selectively expose ferroptosis vulnerabilities in cancer cells.

Although direct inhibition of GPX4 has emerged as a canonical strategy to induce ferroptosis, its clinical translation remains challenging owing to systemic toxicity and poor pharmacological properties^124^. In this context, targeting CoQ_10_ oxidoreductases may represent a more clinically tractable strategy. These enzymes function as druggable metabolic nodes that regulate the size of the ubiquinol pool and thus the capacity of cells to buffer lipid peroxidation. Therapeutic perturbation of the CoQ_10_ redox axis therefore offers an attractive opportunity to sensitize tumors to ferroptotic cell death, particularly when combined with treatments that elevate oxidative stress or lipid peroxidation.

Looking forward, the central challenge is to translate these mechanistic insights into clinically actionable strategies. A key priority will be to define which tumors are truly CoQ–oxidoreductase dependent in vivo and under what environmental constraints this dependency emerges. Mounting evidence suggests that ferroptosis liabilities are frequently imposed by the tumor microenvironment, metastatic niche and nutrient availability, implying that conventional in vitro models may underestimate therapeutic opportunities. Systematic efforts that integrate in vivo functional genetics, spatial metabolomics/lipidomics and microenvironment-informed tumor models will be essential to map where CoQ_10_-based defenses become rate limiting for tumor fitness.

A second priority is to resolve the spatiotemporal logic of CoQ_10_ biology in ferroptosis. CoQ_10_ is synthesized in mitochondria but protects membranes across multiple compartments, raising fundamental questions: How are CoQ pools trafficked, partitioned and maintained across organelles? Which membrane systems act as the initiating sites of lipid peroxidation in distinct contexts, and how do organelle contact sites shape the propagation or containment of ferroptotic signals? Addressing these questions will require new tools to quantify CoQ pool size and redox state with subcellular resolution, as well as experimental platforms that can trace CoQ movement and recycling dynamics in living cells and tissues.

Third, therapeutic development will benefit from a biomarker-driven approach. Rather than treating ferroptosis as a uniform vulnerability, clinical translation will probably succeed where patients can be stratified by (1) the architecture of antioxidant defenses (for example, FSP1/GPX4 balance), (2) the size and distribution of cellular CoQ pools, (3) lipid composition and peroxidation-prone membranes and (4) oncogenic programs that remodel redox metabolism (such as NRF2–KEAP1). In this regard, CoQ_10_ oxidoreductases offer not only drug targets but also diagnostic nodes that report the state of ferroptosis defense. An important next step will be to establish robust biomarkers—molecular, lipidomic and functional—that predict response to CoQ_10_-axis perturbation alone or in combination with standard therapies.

Finally, safety and selectivity considerations will shape the translational path. CoQ_10_ redox cycling supports normal physiology, particularly in tissues with high mitochondrial activity. Thus, future therapeutic strategies should prioritize contexts where tumors exhibit non-oncogene addiction to CoQ_10_-dependent defense and should leverage delivery strategies and dosing regimens that maximize tumor exposure while minimizing systemic liabilities. Combination therapies that modestly lower ferroptosis thresholds—rather than forcibly collapsing antioxidant capacity—may provide a practical route to achieve efficacy with tolerable toxicity.

In sum, the CoQ_10_ oxidoreductase network represents a metabolically grounded and mechanistically precise entry point into ferroptosis-based cancer therapy. As the field advances, a unifying goal will be to convert CoQ_10_ redox biology into predictive biomarkers and rational interventions—transforming ferroptosis from a conceptual vulnerability into a controllable therapeutic modality.
